# Supplementary figures and images for: Diversity, antibacterial and antioxidant activities of fungi associated with Apis cerana
Source: PeerJ. 2025 Aug 1;13:e19762. doi: 10.7717/peerj.19762 (PMC12320804; doi:10.7717/peerj.19762)

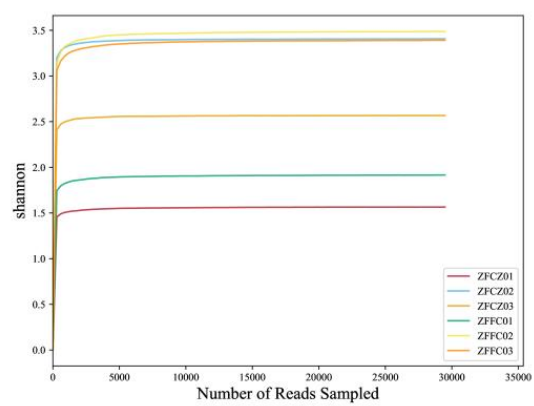

Supplement: Supplemental Information 1 [file peerj-13-19762-s001.pdf]
